# Extremely low neonicotinoid doses alter navigation of pest insects along pheromone plumes

**DOI:** 10.1038/s41598-019-44581-w

**Published:** 2019-05-31

**Authors:** Miguel A. Navarro-Roldán, Carles Amat, Josep Bau, César Gemeno

**Affiliations:** 1 Department of Crop and Forest Sciences, University of Lleida (UdL), 25198 Lleida, Spain; 2 Department of Biosciences, University of Vic – Central University of Catalonia, 08500 Vic, Spain

**Keywords:** Behavioural ecology, Agroecology

## Abstract

The prevailing use of neonicotinoids in pest control has adverse effects on non-target organisms, like honeybees. However, relatively few studies have explored the effect of sublethal neonicotinoid levels on olfactory responses of pest insects, and thus their potential impact on semiochemical surveillance and control methods, such as monitoring or mating disruption. We recently reported that sublethal doses of the neonicotinoid thiacloprid (TIA) had dramatic effects on sex pheromone release in three tortricid moth species. We present now effects of TIA on pheromone detection and, for the first time, navigational responses of pest insects to pheromone sources. TIA delayed and reduced the percentage of males responding in the wind tunnel without analogous alteration of electrophysiological antennal responses. During navigation along an odor plume, treated males exhibited markedly slower flights and, in general, described narrower flight tracks, with an increased susceptibility to wind-induced drift. All these effects increased in a dose-dependent manner starting at LC_0.001_ - which would kill just 10 out of 10^6^ individuals - and revealed an especially pronounced sensitivity in one of the species, *Grapholita molesta*. Our results suggest that minimal neonicotinoid quantities alter chemical communication, and thus could affect the efficacy of semiochemical pest management methods.

## Introduction

Animals moving in fluid media locate odorous resources by upwind orientation, which consists of maintaining their heading against flow^1^. This strategy in itself does not guarantee finding an odor source because flow direction and intensity change through time, producing a shredded and relatively discontinuous odor plume which can be easily lost. To increase the probability of reencountering a lost odor plume, animals orienting upwind typically zigzag across the wind line, and use optic flow to estimate net displacement relative to the ground^2^. Mechanosensory cues appear to contribute to dynamic body control during flight^3^, and even to orientation in specific instances^4^. Thus, animals navigating in a changing flow must sample and integrate chemical, visual and mechanical information, and do so swiftly in order to generate the necessary locomotor responses that will direct them towards an odor source.

Mate finding in moths and other insects is facilitated by sex pheromones, typically produced by females and detected by males from relatively long distances, of perhaps tens or hundreds of meters^5^. The chemical and temporal attributes of the odor plume are detected by highly specific and sensitive pheromone receptor cells located on the antennae, and their responses are integrated in the antennal lobe and higher brain centers^6^. Pheromone perception triggers integrated optomotor anemotaxis and self-steering counterturning programs which guide males to pheromone-releasing females^7^. Sex pheromone research has permitted the development of environmentally-sound semiochemical pest management methods, such as monitoring, mass-trapping and mating disruption (MD), which exploit the response of males to synthetic sex pheromone^8^. While monitoring and mass-trapping rely on male capture with pheromone-baited traps, MD is based on permeation of the treated area with large amounts of pheromone that hinder the male’s ability to locate the emitting female. However, for reasons that are not well understood, MD is effective only in a handful of moth pest species, and its performance is inconsistent in some of the better-controlled ones^9^. Most of the proposed mechanisms of MD involve pheromone detection and response by males^10^, yet females can detect their own pheromone, and so they may also contribute to MD^11^. In addition, there are indications that anthropogenic chemicals, such as residual insecticides, may act as “infodisruptors” interfering with insect olfaction^12^, and thus could compromise semiochemical-based pest surveillance and control methods^13^.

Most commercial insecticides, from DDT in the 1940’s to neonicotinoids in the 1990’s, are neurotoxicants that act on different elements of the nervous system^14^. At the recommended field rates, insecticides cause rapid death, whereas residual doses may induce less evident sublethal effects, with consequences in pest control that remain largely unexplored^12,15–17^. Neonicotinoids are nicotinic-acetyl choline receptor (nAChR) agonists and affect nerve transmission in the predominantly cholinergic synapses of the insect’s CNS^18^. Studies on the sublethal effects of neonicotinoids have focused mainly on honeybees, where they cause several deleterious effects, including a reduced ability to navigate back to the hive^19^. In contrast, few studies have tested the sublethal effects of neonicotinoids on pest insects^20–24^, and none, as far as we know, have examined if neonicotinoids affect navigation manoeuvres of pest insects to odour sources.

We recently found that topical application of the neonicotinoid thiacloprid (TIA) substantially reduced pheromone release behaviour in females of three tortricid moth species [*Cydia pomonella* (L.), *Grapholita molesta* (Busck) and *Lobesia botrana* (Denis & Schiffermüller); from here on “CP”, “GM” and “LB”, respectively], starting at the lethal concentration LC_0.001_ which kills only 10 in 10^6^ individuals^25,22^. In the present study, we explore the influence of TIA on male perception of, and response and orientation to sex pheromone. The behavioral response of males treated with extremely low doses of TIA is compared to the performance of control males in wind tunnel assays. Video-recorded flight tracks are analysed, considering several parameters related to flight speed and direction with respect to the wind line, including thrust and deviation from intended course due to the airflow. In addition, electroantennograms (EAG) are used to assess the effect of TIA on pheromone detection at the antennal level. We speculate on the consequences of our findings in the context of pest management programs that combine the use of insecticides and sex pheromones.

## Results

### Wind tunnel

**Figure 1:**
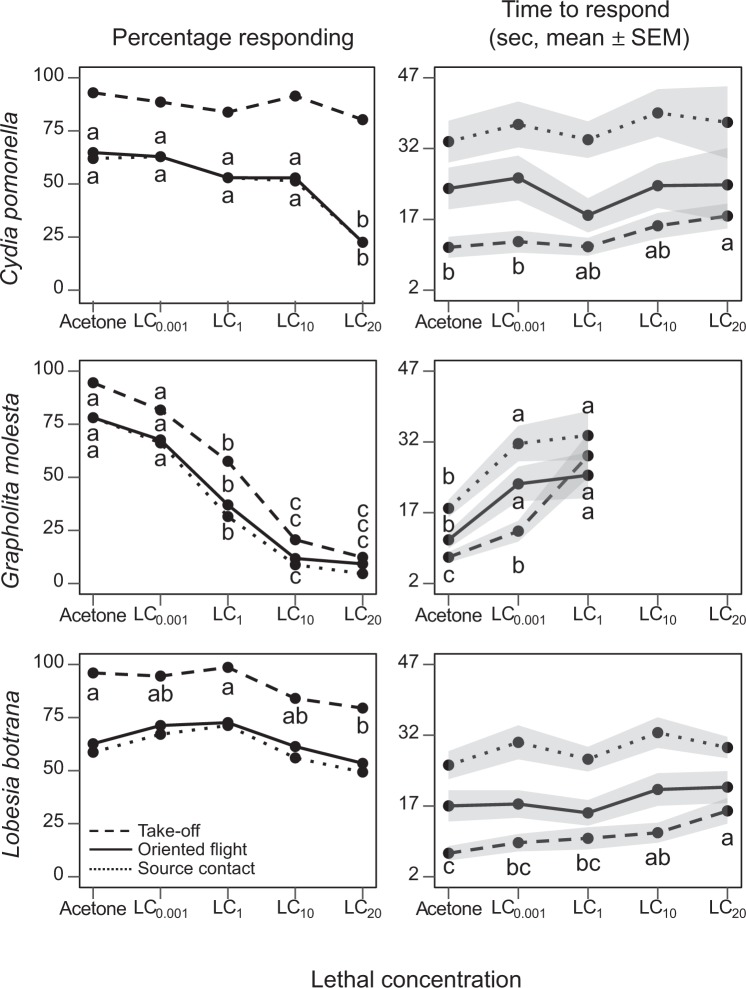
Effect of thiacloprid (TIA) on percentage of *C. pomonella*, *G. molesta* and *L. botrana* males responding (taking flight, orienting upwind, and contacting the stimulus) to sex pheromone in wind tunnel (left), and the time to start these behaviours (right). Males treated with acetone or 4 lethal concentrations of TIA (LC_0.001_ to LC_20_) were released individually and observed for 2 minutes, or until contact with the pheromone dispenser. The shadowed areas around the mean trend line in the times graph indicate ± SEM. Different letters indicate significant differences among TIA LCs within a given behavioural category (Tukey’s test after GLM or ANOVA, P < 0.05).

Percentages of mortality after insecticide treatment (Supplementary Table S2) roughly corresponded with typified dose-mortality curves^25^. Sublethal doses of TIA reduced significantly the percentage of GM and LB taking flight, and the percentage of CP and GM orienting to and contacting with the pheromone source (Fig. 1, Supplementary Table S3). This disruptive effect was notable in GM, moderate in CP and limited in LB. It is noteworthy that for GM it was already significant at the second lowest concentration LC_1_, and at the highest dose it reduced contact by 94% compared with the acetone control. For LB and CP only the highest TIA concentration (LC_20_) produced a significant effect, with a maximum contact reduction of 64% in CP. TIA application delayed significantly the onset of all behavioural categories in GM and the time to take flight in CP and LB (Fig. 1, Supplementary Table S3). Such delays were significant even at the lowest TIA dose (LC_0.001_) in GM, but only at the highest doses in CP and LB (Fig. 1, Supplementary Table S3).

**Figure 2:**
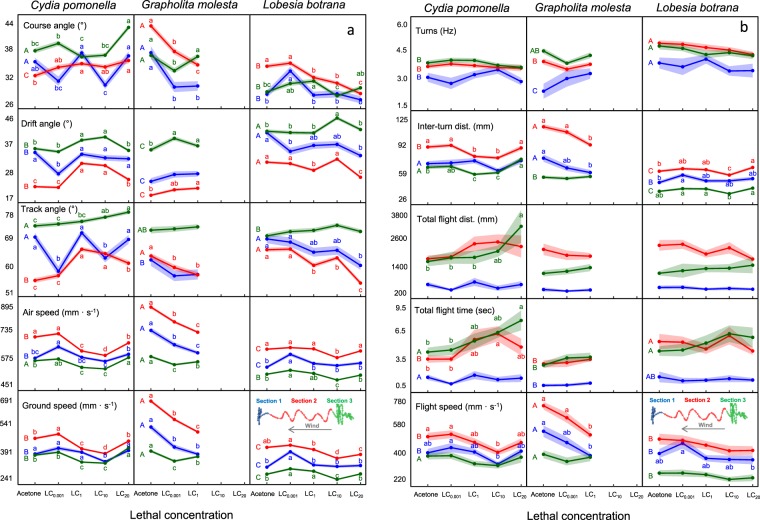
Effect of thiacloprid (TIA) on flight track parameters of males of *C. pomonella*, *G. molesta* and *L. botrana* responding to sex pheromone in the wind tunnel. (**a**) Triangle of velocities parameters. (**b**) Flight track parameters. Males treated with acetone or 4 lethal concentrations of TIA (LC_0.001_ to LC_20_) were released individually and observed for 2 minutes or until contact with the pheromone dispenser. The concentrations LC_10_ and LC_20_ were omitted in *G. molesta* due to insufficient number of responding males. The flight track is divided in three sections (inserts) corresponding with the distance to the pheromone source: 130–100 cm (section 1, flight initiation); 100–30 cm (section 2, oriented flight); 30–0 cm (section 3, pre-contact), and each section was analysed individually. The shadowed areas around the mean trend line indicate ± SEM. Different small letters indicate significant differences among TIA LCs within a given behavioural category and flight track section. Different capital letters indicate significant differences among species for individuals treated with control acetone (Tukey’s test after ANOVA, P < 0.05).

TIA significantly affected track parameters in all three species (Fig. 2a,b, Supplementary Table S4, Supplementary Videos). Although the flight parameters varied across wind tunnel track sections (flight initiation, oriented flight and pre-contact; see Fig. 2a,b), the general effect of TIA was similar among them, so here we will focus our attention on the oriented flight section (section 2), where most upwind displacement takes place. In general, males treated with TIA were significantly more susceptible to wind drift (had higher drift angle), in accordance with a significant reduction in their actual and apparent speeds (airspeed and groundspeed, respectively) (Fig. 2a). With TIA the intended flight direction with respect to wind (course angle) increased significantly in CP and decreased in the other two species, as did the apparent angle of flight with respect to the wind line (track angle) (Fig. 2a). Total flight time increased and average flight speed decreased in CP, but no changes were observed in the other two species (Fig. 2b). TIA significantly reduced inter-turn distance in CP and GM, and so their flight tracks were narrower, except at the highest dose where LB presented longer, but straighter upwind (i.e., lower track angles), inter-turn legs. On the other hand, turning frequency was maintained constant in all species, indicating that the self-steered counterturning mechanism was not altered by insecticide exposure (Fig. 2b). With regard to differences between track sections, in section 2 males flew significantly faster, straighter and exhibited wider turns, while in section 3 they reduced their flight speed, flew more perpendicular to the wind line and performed more turns as they approached the pheromone (Fig. 2a,b, Supplementary Table S4, Supplementary Videos). Values in section 1 were intermediate between those of sections 2 and 3.

Comparison among species using control (acetone-treated) individuals showed acute differences in flight track parameters (Fig. 2a,b, Supplementary Table S5, Supplementary Videos). In section 2, where most upwind displacement occurs, it is clear that GM flew faster, experienced less drift and performed wider turns than the other two species. In contrast, LB exhibited higher turn frequency and shorter inter-turn distances than the other two species.

### Electroantennograms

**Figure 3:**
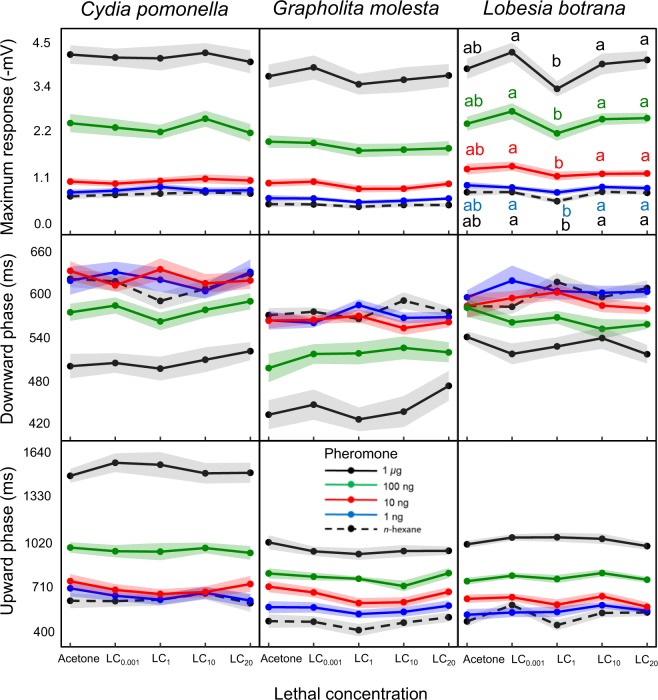
Effect of thiacloprid (TIA) on electroantennogram (EAG) responses of males of *C. pomonella*, *G. molesta* and *L. botrana* to sex pheromone stimulation. Males treated with acetone or 4 lethal concentrations of TIA (LC_0.001_ to LC_20_) were stimulated with *n*-hexane and 3 concentrations (1 ng to 1 *µ*g) of the major pheromone components of *C. pomonella* (*E,E*-8,10-12:OH), *G. molesta* (*Z*8-12:Ac) and *L. botrana* (*E*,*Z*-7,9-12:Ac). Three EAG parameters were measured: maximum response, and the duration of the downward and upward phases. The only significant TIA effect was a reduction of maximum response with LC_1_ in *L. botrana*. For each pheromone dose, different letters indicate significant differences among TIA doses, Tukey’s test after ANOVA (P < 0.05).

ANOVA analysis of the TIA tests revealed a significant effect of pheromone concentration in all cases (Fig. 3, Supplementary Table S6). Nowhere the interaction between pheromone concentration and TIA was significant. In addition, TIA affected maximum EAG response in LB (Supplementary Table S6). Tukey’s pairwise comparisons showed a reduction of LC_1_ relative to the other TIA doses or the acetone control (Supplementary Table S7). When the pairwise tests were done for each individual pheromone dose the result was similar, except that LC_1_ and the acetone control did not differ from each other (Fig. 3, Supplementary Table S7). TIA also had a significant effect on the upward phase of the EAG (i.e., the time from maximum response to baseline voltage) in GM and LB (Supplementary Table S6). In GM, the Tukey’s pairwise test indicated a significant effect of LC_1_, but this effect disappeared when the comparison was done for each pheromone dose individually (Supplementary Table S7). In LB, the Tukey’s test did not discriminate among TIA levels (Supplementary Table S7), although TIA was significant in the model (Supplementary Table S6). In summary, the most relevant effect of TIA was a reduction of maximal negative voltage in LB with LC_1_, but not with higher or lower doses (Fig. 3).

## Discussion

In this study, we show that the neonicotinoid insecticide thiacloprid reduced the ability of male moths to normally respond and orient to pheromone plumes in the wind tunnel after application of insecticide concentrations as low as LC_0.001_. It is unlikely that this effect was caused by a dysfunction in the peripheral sensory system, as there was only a minor EAG impairment that was not consistent across TIA doses and did not conform with the behavioural changes. Considering that TIA alters synaptic transmission^21^, an effect at the CNS level is more likely. The percentage of responding males decreased with increasing TIA concentration and their flights became slower, differently angled in relation to the wind line, and more susceptible to wind drift (depending on the species), all of which could render them less capable of odour source location. Together with our previous finding that similar low doses of TIA reduce pheromone release behaviour in females, the hypothesis that residual neonicotinoid levels could affect semiochemical pest-management control in these species is reinforced, especially when synthetic lure-finding is involved (i.e. monitoring or attract and kill).

TIA acts on nAChRs, which in insects are located mainly in the CNS^26^. Although the activity of neonicotinoids on nAChRs is well established^18^, almost no studies have investigated the ensuing physiological and behavioural effects. One exception is a recent study which demonstrates that ingestion of low doses of the neonicotinoid clothianidin alters the response of antennal lobe (AL) interneurons in *Agrotis ipsilon* (Hufnagel)^21^, and that these changes correspond with changes in the percentage of males responding to sex pheromone in the wind tunnel^27^. Suboptimal pheromone quantity and composition alters flight track parameters of *G. molesta* in ways that resemble the changes that we observed with TIA (i.e., reduced air speed, increased drift angle and narrower inter-turn distance)^28,29^. This supports the hypothesis that TIA altered pheromone perception in our test insects.

Sublethal doses of the neonicotinoid imidacloprid impair the motion-sensitive visual network of *Locusta migratoria* L. affecting their escape response^23^. Similarly, sublethal exposure to imidacloprid alters the motion-detection neural system of a hoverfly pollinator^30^. Since visual flow across the retina provides essential cues for regulating orthokinetic (movement) and taxis (steering) components of the flight tracks^31^, a potential effect of TIA on the visual system could explain some of the effects that we have observed. On the other hand, the reduction in the number of males taking flight, together with an increased wind-induced drift angle, suggest that TIA could have attenuated male muscular activity. Whereas neuromuscular transmission in vertebrates and in several invertebrates is cholinergic, synaptic transmission at the insect neuromuscular junction is glutamatergic^32^, so a direct effect of TIA on the motor system is unlikely, but an alteration of the muscular system via the CNS is still possible. A potential neuronal equivalent of the zig-zag pattern of moths is found in an alternating “on and off” activity of two nerve cords that descend from the brain to the suboesophageal ganglium in the non-flying moth *Bombix mori* L.^33^. This neural system could be a target subject for future studies of neonicotinoid effects on flight performance.

Since insects have no, that we currently know, olfactory synapses or nAChRs in the antenna^34^, it is not surprising that TIA barely affected EAG responses. The only significant effect was a reduction of maximum response in LB with the intermediate dose LC_1_. Furthermore, this effect was not dose-dependent and did not correlate with the behavioural effects observed in the wind tunnel. A similar lack of effect of the neonicotinoid clothianidin on EAG responses in the black cutworm moth, *A. ipsilon* has been reported^21^. Although direct effect of TIA on EAG activity was unlikely, alternative routes of action are still possible. For example, metabolic by-products of TIA from detoxification enzymes^35–37^ could interact with the chemosensory proteins that mediate olfactory processes, as has been reported in other insects with neonicotinoids^38,39^. On the other hand, sensory parameters such as the ability to resolve odor pulses, which is an essential aspect of odor guided-behavior in moths^40^, were not investigated here, so we cannot completely rule out a larger impact of TIA at the peripheral level.

At sublethal levels, insecticide effects on mating behavior^41–44^ could have a direct impact on pest population levels. However, the potential effect of residual insecticide on semiochemical control methods remains speculative^13,45^. The extent of such effects can differ depending on the insect species, but also on the specific type of control mechanism, as existing methodologies rely on different modes of action. Approaches like pest monitoring, lure and kill or mass trapping, aim at attracting the male to pheromone-baited traps, while the role of synthetic pheromone in MD methods is to disorient or mislead the male, rendering it unable to locate the females or, by extension, any emission point-source. In the context of MD, two major mechanism types have been proposed, competitive (CMD) which depends on the relative attraction of males to either MD pheromone dispensers or females, with no sensory impairment in either males (or females), and non-competitive (NCMD) where sensory disability of males (or females) reduces sexual activity^10^. We speculate that the negative effect of TIA on female calling behavior^22^ could increase the competiveness of pheromone dispensers relative to females, which should potentiate CMD and have no net effect on NCMD. On the other hand, a negative impact of TIA on males could enhance NCMD, while it should have no effect on CMD. This argument can also be applied to control methods based on male attraction to synthetic lures such as monitoring, attract and kill and mass trapping. Relative attractiveness of pheromone traps with respect to females could be accentuated if TIA reduced female calling behavior. Similarly, a weakened male response due to TIA would render females (which probably have relatively lower pheromone emission rates than pheromone trap lures) less attractive to males than to synthetic attractants. In absolute terms, the same reduced male response to synthetic lures could have an impact on the effectiveness of surveillance methods. Computer simulations on forest pests reveal that low trap efficiencies (applicable in this case to decreased male responsiveness) cause low-level populations to remain undetected under standard surveillance conditions^46^. This can significantly affect the efficacy of these pheromone-based methods, especially considering that free-ranging males would often encounter pheromone plumes further downwind in the field than in a wind-tunnel^5^, so the effect of sublethal TIA intoxication could still be amplified when considering source location efficiency in the field.

## Materials and Methods

### Insects and insecticide application

Insects from insecticide-susceptible colonies were reared on artificial diet at 25 ± 1 °C under a 16:8 h light:dark photoregime. Colony origin and further rearing descriptions have been detailed elsewhere^22^.

Four lethal concentrations (LC_0.001_, LC_1_, LC_10_ and LC_20_) of TIA insecticide [PESTANAL®, analytical standard, ≈100% (a.i.), diluted in acetone] were tested following previous dose-mortality calibrations^25^. The concentrations (ng a.i./*µ*l) applied to CP, GM, and LB, respectively, were: LC_0.001_ (0.61, 4.88 and 32.29), LC_1_ (4.24, 15.05 and 164.66), LC_10_ (12.04, 27.63 and 396.19) and LC_20_ (18.70, 35.68 and 573.40). Control insects were treated with acetone from the same stock used to make the insecticide dilutions. Within 24 hours after emergence, males received 1-*µ*l topical application of a test treatment on the ventral side of the thorax after 10-sec CO_2_ anaesthesia, and were placed in 150-ml polypropylene bottles (3 to 10 individuals per bottle) with 10% sugar solution. Insecticide effect was assessed 24 h post-treatment and scored as alive if apparently unaffected, moribund if clearly affected but still moving, or dead if immobile. Mortality was estimated with the sum of moribund and dead insects. Alive insects were used in the experiments on the same day (age 24–48 h).

### Pheromone stimuli

Sex pheromone compounds (Pherobank, The Netherlands, purity ≥99%) were diluted in *n*-hexane. For GM the 3-component pheromone blend [(*Z*)-8-Dodecenyl acetate (*Z*8-12:Ac), (*E*)-8-Dodecenyl acetate (*E*8-12:Ac), and (*Z*)-8-Dodecen-1-ol (*Z*8-12:OH) in a 100:6:10 ratio]^47^ was used in wind tunnel tests, while EAG tests were performed with just the major pheromone compound, *Z*8-12:Ac. For CP we used codlemone [(*E*,*E*)-8,10-Dodecadien-1-ol; *E*,*E*-8,10-12:OH] in both wind tunnel and EAG tests. Due to the relatively poor behavioural response of LB to synthetic blends^48^, for this species we used pheromone gland extracts in the wind tunnel assay (following methodology described previously)^22^, and the major pheromone compound, [(E,Z)-7,9-Dodecenyl acetate; *E*,*Z*-7,9-12:Ac] in EAG tests.

### Flight tunnel

Flight experiments were conducted in a 170 × 45 × 45 cm glass wind-tunnel with a solid black floor at a temperature of 23.5 ± 1 °C and a 0.32 m · s^−1^ air flow (see^49^ for further wind-tunnel description). Two 25-watt incandescent bulbs covered with a white fabric for diffusion provided illumination 150 cm above the insect flight plane. Visible light intensities (mean ± SEM) were set at 20.2 ± 0.2 lux during photophase and 3.1 ± 0.1 lux during scotophase. Image contrast of both daytime and night time flight track recordings was enhanced by means of four lateral infrared illuminators (peak frequency 850-nm) equipped with 96 LED lights each, of either 30° or 60° beam angles (Scene Electronics, Kowloon, China).

The odour source consisted of 100 ng of pheromone blend (GM and CP) or 1 female equivalent (LB) applied in 10 *μ*l loads onto 10 × 15 mm *n*-hexane-rinsed filter papers (Whatman® No. 1, Sigma-Aldrich), readily prepared every day and kept until use in a sealed vial after solvent evaporation (10 min). The filter paper stood flat against the wind on a 12.5 cm-high metal-wire platform and males were released from a second platform positioned 1.30 m downwind.

Between 65 and 75 males per treatment were assayed in randomized blocks and the onset times of flight initiation (take flight), upwind zigzagging (oriented flight), and source contact were recorded. Males habituated in individual tubes in the wind tunnel room for 30 min prior to flight tests, which were carried out during the peak female-calling period of each species: 14^th^–16^th^ hours of the photophase for GM, 2^nd^–4^th^ hours into the scotophase for CP and the 1^st^–2^nd^ hours of the scotophase for LB^22^. Males were observed for 2-min to obtain the percentages and onset times of response. Those initiating flight within a 2-min observation period were video-recorded with a CCTV camera (DINION IP 5000 HD with 5.0–50 mm f/1.4 objective, Bosch Security Systems) positioned 140 cm above the flight plane. The captured area had minimal angular distortion and spanned the width of the tunnel from the pheromone dispenser up to 129 cm downwind, almost at the point of insect release, (Supplementary Fig. S1, Supplementary Videos). 2D track coordinates were extracted with Blender v2.78 (www.blender.org) from 25 fps video sequences. Triangle of velocities and other track parameters were calculated using a custom flight track analysis software (Supplementary Table S1, Fig. S2). Twenty videos were obtained for each TIA dose and acetone control except for LC_10_ and LC_20_ in GM, where the number of responding males was too low for statistical analysis.

### Electroantennograms

Males were restrained with a modified alligator clip (Supplementary Fig. S3) after 10 sec CO_2_ anaesthesia. A glass capillary containing a gold wire electrode filled with 1% NaCl solution was inserted in the mouth (reference electrode) and another contacted the trimmed tip of the left antenna (recording electrode). The signal from the recording electrode was pre-amplified (10 × gain, Universal Single Ended Probe, Syntech, Germany), filtered (0.1 Hz–1 kHz), digitized (IDAC-4, Syntech), recorded and analyzed (AutoSpike v.3.9, Syntech).

Pheromone stimuli were prepared as in wind-tunnel experiments, introduced into disposable blue plastic 1000 *μ*l pipette tips and kept in sealed glass tubes. A 0.6 l · min^−1^ flow of charcoal-filtered and humidified air (CS-55, Syntech) blew continuously over the insect preparation through an 8-mm internal diameter steel tube placed 2 cm from the preparation. The pipette tip containing the odour stimulus was inserted in the tube 11 cm from the flow outlet and 0.5 s puffs of 0.3 l · min^−1^ were delivered with a concomitant and equal decrease of the continuous flow to maintain a constant flow on the antenna and minimize non-chemical responses. Stimuli were applied in increasing order of concentration (*n*-hexane, and major pheromone component at 1 ng, 10 ng, 100 ng, 1 µg, 10 µg and 100 µg load on filter paper), allowing full antennal recovery to baseline between puffs. A dose-response curve with acetone-treated insects (N = 20, Supplementary Fig. S4) indicated that the 1 ng to 1 *µ*g concentration range was optimal (i.e., below saturation) for use in the TIA tests (N = 20). EAG traces typically consist of a relatively large negative voltage wave that is usually followed by a relatively smaller positive voltage wave. The three EAG parameters analysed were the maximum voltage of the negative wave (i.e, maximum response), the time from baseline voltage to maximum response (i.e., downward phase), and the time from maximum response to baseline voltage (i.e., upward phase) (Fig. S5).

### Statistical analyses

Statistical analyses were run in R software^50^. Continuous data were analysed with linear models (LM), and discrete data (percentages) with generalized linear models (GLM) using the binomial function. To analyse the percentage of responding males and the time to respond the statistical models, one per species, contained insecticide concentration as the only model term. For the analysis of track parameters, we took into consideration the distance of the insect to the odour source, which is known to affect flight track parameters^51^. Recorded tracks were split in three sections: (1) flight initiation (130 to 100 cm downwind = section 1), (2) oriented flight (100 to 30 cm = section 2), and (3) pre-contact (30 to 0 cm downwind = section 3). Flight track models included insecticide concentration, flight track section, and their interaction. For inter-species comparison of track parameters, only control (acetone) individuals were used and, in this case, each flight track section was analysed individually, and species was the only model term. To test the effect of TIA, pheromone concentration and their interaction on EAG responses, one model for each species and EAG parameter (maximum response, and duration of the downward and upward phases) was run. Data transformation [log (x + 1) or sqtr (x + 1)] was performed whenever needed to improve the fit of model residuals. Models were ranked based on a likelihood ratio test. Treatment means were compared with Tukey’s test, except for mortality where we used Fisher’s Exact Test with Bonferroni corrections. Throughout the text statistical analyses are reported as significant when P value < 0.05. P values are shown in the statistical tables located in the supplementary file. All the data obtained during the study, as well as the R scripts used in the statistical analyses, are available at the University of Lleida repository [http://hdl.handle.net/10459.1/65433].

## Supplementary information

Supplementary Video

Supplementary file

## Data Availability

Original data and R codes for statistical analysis are available at the University of Lleida repository, http://hdl.handle.net/10459.1/65433.
