# Supplementary material for: Extremely low neonicotinoid doses alter navigation of pest insects along pheromone plumes
Source: Sci Rep. 2019 May 31;9:8150. doi: 10.1038/s41598-019-44581-w (PMC6544627; doi:10.1038/s41598-019-44581-w)
Supplement: Supplementary file 2 — Supplementary file [file 41598_2019_44581_MOESM2_ESM.pdf]

Extremely low neonicotinoid doses alter navigation of pest insects along pheromone plumes

Miguel A. Navarro-Roldán, Carles Amat, Josep Bau and César Gemeno

Supplementary material

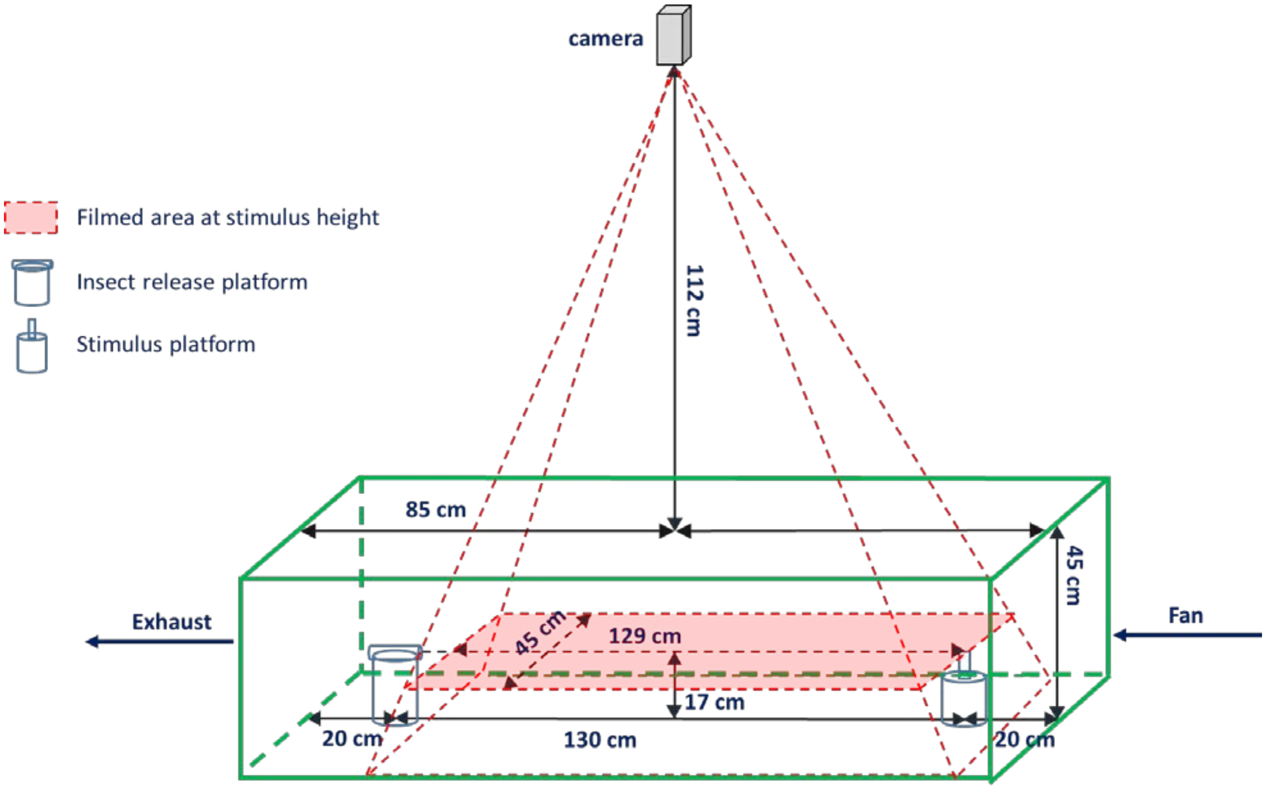

Figure S1. Wind tunnel plan.

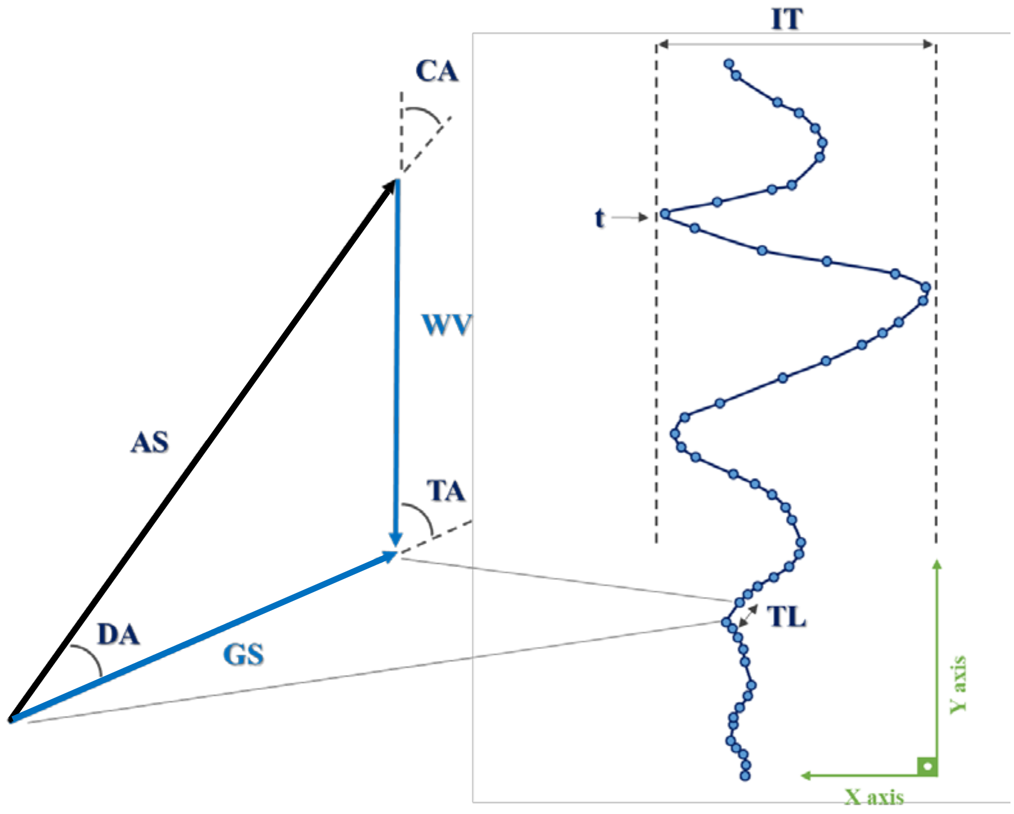

Figure S2. Flight track and derived track parameters. See Table S1 for description.

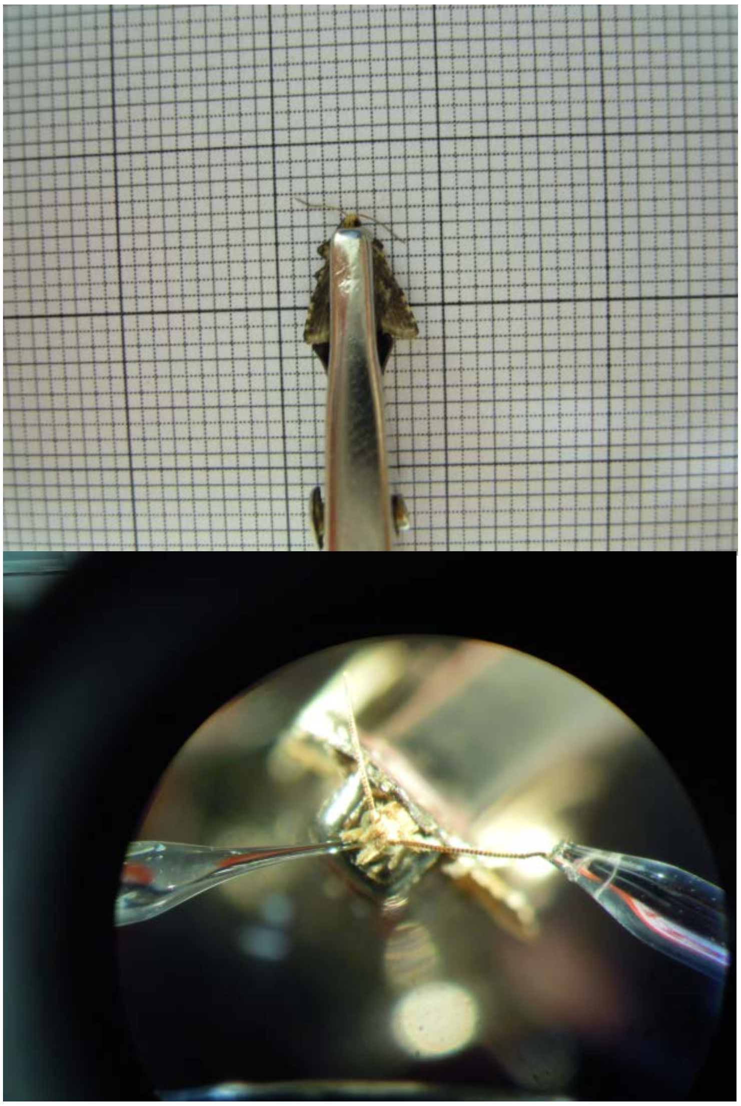

Figure S3. Alligator clip to hold moths (top) and close up of EAG setup (bottom; reference electrode on the left, recording electrode on the right, *Lobesia botrana* both).

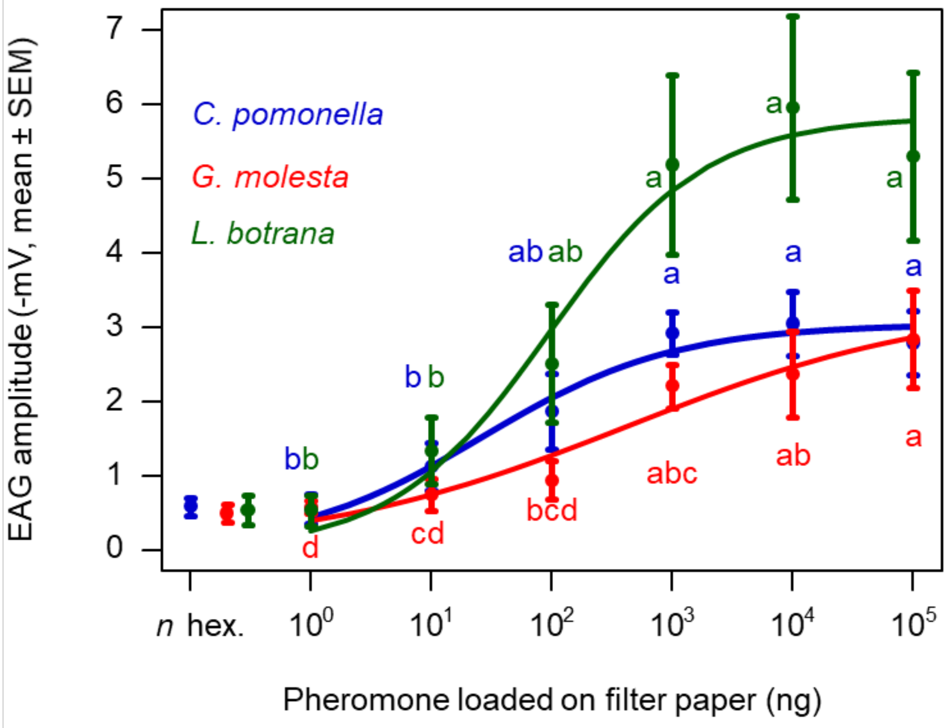

Figure S4. Effect of major pheromone compound load on EAG amplitude of control (acetone) insects.

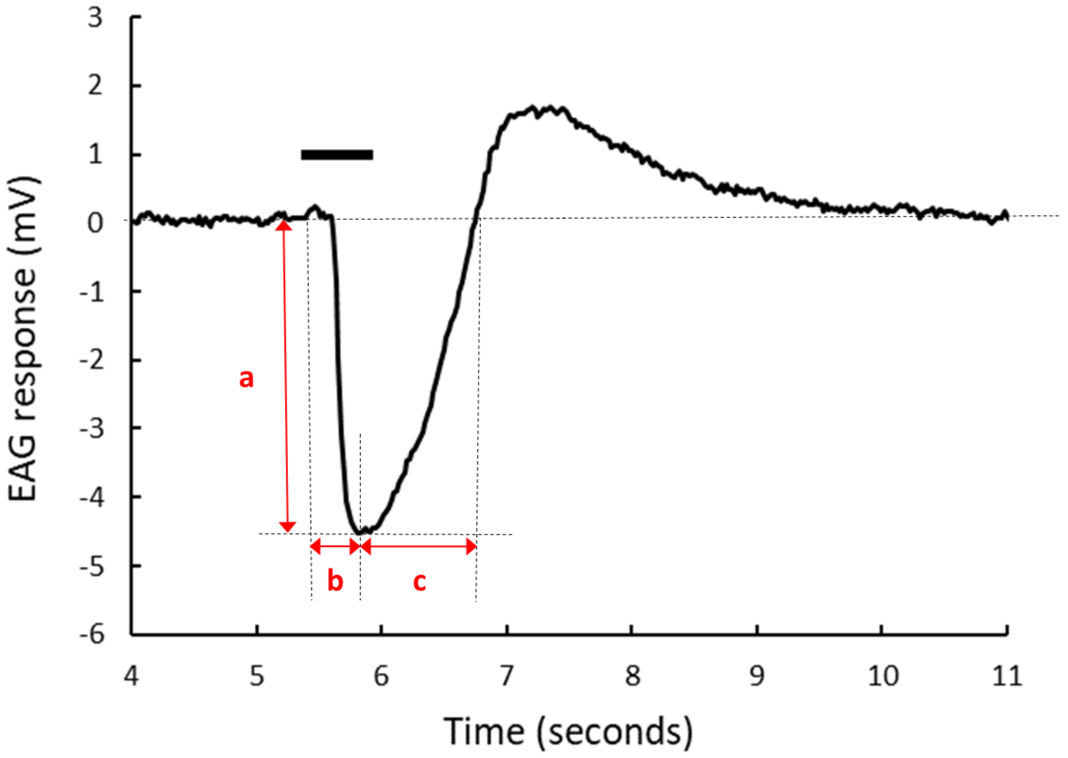

Figure S5. Representative EAG trace showing the parameters analysed in the TIA test. This trace is from a male of *Lobesia botrana* (individual #8 in the dataset) treated with TIA LC_{20} and stimulated with 1 μg pheromone (*E*,*Z*-7,9-12:Ac) for 500 ms (solid black horizontal bar above the trace). The three parameters analysed were: **a)** maximum response (-4.54 mV in this example), **b)** time from baseline voltage (≈ 0 mV) to maximum response (i.e., downward phase, 0.425 s in this example), and **c)** time from maximum response to baseline voltage (i.e., upward phase, 0.94 s in this example). The beginning of parameter "b" corresponded with a small positive potential just after onset stimulation that was visually determined by the observer (it is relatively small in this trace). The upward phase (c) moved up into positive voltages after reaching the baseline, but this part of the trace was variable among species and pheromone doses and so it was not included in the analysis.

Table S1. Flight track parameters analysed in the study. See Figure S2 for graphical representation.

Parameter	Abbr.	Units	Description
Ground speed	GS	$mm·s^{-1}$	It is the length of the track length (TL, movement of the insect between two video frames, in mm) divided by the time between two video frames (1/25 fps). *It represents the actual (recorded) speed, or "thrust", of the insect between two frames.*
Air speed	AS	$mm·s^{-1}$	It is the length of the vector that goes from the beginning of the TL vector to the beginning of the wind speed vector (WV, the actual wind speed in $mm·s^{-1}$) divided by the time between two video frames (1/25 fps). *It represents the "intended" thrust of the insect.*
Wind Vector	WV	$mm·s^{-1}$	The actual wind speed. Variable controlled by the observer in Wind Tunnel
Track angle	TA	Degrees	Angle formed between WV and TL vectors. It is the actual angle of displacement of the insect between two frames relative to the direction of the wind at that moment. *It represents the actual (recorded) direction of displacement of the insect.*
Course angle	Course angle	Degrees	It is the angle between AS and WV. *It represents the direction that the insect "intended" to take with respect the wind direction, before being displaced by the wind, i.e., before wind drift.*
Drift angle	DA	Degrees	Angle between AS and GS. *It represents how much displacement has exerted the wind on the insect intended trajectory*
Number of turns	T		Whenever the insect changed its direction by more than a set number of degrees it was considered a turn (t). In our study we used the number of turns per second, which is the total number of turns (T) divided by time. *Turn frequency appears to be one of the most species-specific and invariable flight track parameters in moths, one that is self generated.*
Intern-turn reversal	IT	mm	Distance between two turns in the Y-axis. It is a measurement of the amplitude of the insect displacement relative to the centre of the wind tunnel. *It shows how wide the zig-zagging flight is, the width of the flight track*
Total flight length	FL	mm	Total distance covered by the insect during a given section of the flight track. It is the sum of the individual TL vectors in that section.
Total flight duration	FD	s	Time needed by the insect to complete the FL
Total flight velocity	FV	$mm·s^{-1}$	= FL/FD

Table S2. Mortality caused by sublethal doses of thiacloprid on males used in wind tunnel and EAG experiments. Different letters in same column indicate significant differences among treatments in each species (P<0.05, Fisher Exact test with Bonferroni correction after GLM).

Species	Treatment	Thiacloprid (ng)	Wind tunnel test			EAG test		
			N	% dead 24 h		N	% dead 24 h	
Cydia pomonella	Acetone	0	109	0.00	b	54	0.00	b
	$LC_{0.001}$	0.61	110	0.00	b	54	0.00	b
	LC_1	4.24	112	1.79	ab	54	0.00	b
	LC_{10}	12.04	117	5.13	ab	56	0.00	b
	LC_{20}	18.70	125	10.40	a	59	23.73	a
Grapholita molesta	Acetone	0	73	0.00	b	61	0.00	c
	$LC_{0.001}$	4.88	73	1.37	b	61	0.00	c
	LC_1	15.05	73	0.00	b	61	6.55	bc
	LC_{10}	27.63	75	9.33	ab	61	18.03	b
	LC_{20}	35.68	79	17.72	a	63	30.16	a
Lobesia botrana	Acetone	0	75	0.00	b	62	0.00	
	$LC_{0.001}$	32.29	74	0.00	b	62	0.00	
	LC_1	164.66	75	2.67	ab	63	0.00	
	LC_{10}	396.19	80	6.25	ab	63	4.76	
	LC_{20}	573.40	81	9.87	a	63	6.35	

Table S3. GLM and ANOVA results for the effect of TIA on the percentages of males responding (A) and time to respond (B) in the wind tunnel. Percentages, means and SEMs, and significant differences, shown in Figure 1.

**A**						
**Species**	**Behaviour**	**Df**	**Deviance**	**Resid. Df**	**Resid. Dev**	**Pr(>Chi)**
	Take flight	4	7.14	345	257.57	0.1285
C. pomonella	Oriented flight	4	33.92	345	451.10	7.727e-07
	Source contact	4	31.73	345	453.46	2.174e-06
	Take flight	4	166.49	345	315.79	< 2.2e-16
G. molesta	Oriented flight	4	123.86	345	351.69	< 2.2e-16
	Source contact	4	144.18	345	323.49	< 2.2e-16
	Take flight	4	24.58	364	206.87	6.116e-05
L. botrana	Oriented flight	4	7.87	364	473.38	0.0966
	Source contact	4	9.50	364	485.85	0.0497

**B**						
**Species**	**Behaviour**	**Df**	**Sum Sq**	**Mean Sq**	**F**	**Pr(>F)**
	Take flight	4	20.29	5.07	3.36	0.0103
C. pomonella	Oriented flight	4	1.83	0.46	0.31	0.8712
	Source contact	4	1.43	0.36	0.79	0.5331
	Take flight	2	37.56	18.79	19.62	2.24e-08
G. molesta	Oriented flight	2	17.44	8.72	10.49	6.039e-05
	Source contact	2	8.62	4.31	12.62	1.029e-05
	Take flight	4	20.29	5.07	3.36	0.0103
L. botrana	Oriented flight	4	5.62	1.40	0.98	0.4167
	Source contact	4	2.45	0.61	1.61	0.1723

Table S4. ANOVA results for the effect of insecticide and wind tunnel section on flight track parameters. Means, SEMs and significant differences shown in Figure 2.

Species	Parameter [a]	Variable [b]	Df	Sum Sq	Mean Sq	F	Pr(>F)
C. pomonella	TA	TIA	4	528	132.02	24.27	< 2.2e-16
		sect	2	4962	2481.05	456.04	< 2.2e-16
		TIA*sect	8	540	67.44	12.40	< 2.2e-16
	CA	TIA	4	405	101.19	23.24	< 2.2e-16
		sect	2	797	398.45	91.50	< 2.2e-16
		TIA*sect	8	323	40.34	9.26	7.754e-13
	DA	TIA	4	907	226.80	71.20	< 2.2e-16
		sect	2	4698	2349.21	737.44	< 2.2e-16
		TIA*sect	8	286	35.69	11.20	5.883e-16
	GS	TIA	4	14766	3691.40	113.90	< 2.2e-16
		sect	2	21501	10750.40	331.69	< 2.2e-16
		TIA*sect	8	2620	327.60	10.11	3.468e-14
	AS	TIA	4	9366	2341.50	136.59	< 2.2e-16
		sect	2	24645	12322.60	718.82	< 2.2e-16
		TIA*sect	8	2258	282.20	16.46	< 2.2e-16
	T	TIA	4	0.1826	0.05	1.07	0.3719
		sect	2	2028	1.14	26.74	2.532e-11
		TIA*sect	8	0.59	0.07	1.72	0.0930
	IT	TIA	4	572.00	142.92	18.51	4.324e-15
		sect	2	2702.00	1350.88	174.92	< 2e-16
		TIA*sect	8	158.00	19.79	2.56	0.0087
	FL	TIA	4	1414.10	353.50	3.04	0.0179
		sect	2	28950.40	14475.20	124.38	< 2e-16
		TIA*sect	8	1704.70	213.1	1.83	0.0714
	FD	TIA	4	7.09	1.77	6.36	6.585e-05
		sect	2	71.73	35.86	128.78	< 2.2e-16
		TIA*sect	8	4.47	0.56	2.01	0.0457
	FV	TIA	4	194.81	48.70	7.87	5.145e-06
		sect	2	426.29	213.15	34.45	4.715e-14
		TIA*sect	8	23.82	2.98	0.48	0.8690
G. molesta	TA	TIA	2	34	17.23	2.77	0.0626
		sect	2	1733	866.32	139.38	< 2.2e-16
		TIA*sect	4	124	30.95	4.98	0.0005
	CA	TIA	2	324	161.99	34.78	8.746e-16
		sect	2	297	148.66	31.92	1.504e-14
		TIA*sect	4	230	57.38	12.32	5.367e-10
	DA	TIA	2	167	83.53	23.69	5.428e-11
		sect	2	4101	2050.41	581.45	< 2.2e-16
		TIA*sect	4	21	5.35	1.52	0.1939
	GS	TIA	2	11994	5997	163.56	< 2.2e-16
		sect	2	63471	31735	865.57	< 2.2e-16
		TIA*sect	4	3800	950	25.91	< 2.2e-16
	AS	TIA	2	5658	2829.00	121.05	< 2.2e-16
		sect	2	45651	22825.70	976.67	< 2.2e-16
		TIA*sect	4	1679	419.8	17.96	1.033e-14
		TIA	2	0.56	0.28	1.56	0.2128

	T	sect	2	8.16	4.08	22.75	1.731e-09
		TIA*sect	4	1.84	0.46	2.57	0.0399
	IT	TIA	2	173.20	86.60	11.42	1.174e-05
		sect	2	6555.30	3277.60	432.25	< 2e-16
		TIA*sect	4	186.00	46.50	6.13	6.666e-05
	FL	TIA	2	32.1	16.00	0.25	0.7818
		sect	2	18305.70	9152.80	140.78	< 2e-16
		TIA*sect	4	177.7	44.40	0.68	0.60
	FD	TIA	2	0.96	0.48	2.93	0.0562
		sect	2	32.02	16.01	97.27	< 2e-16
		TIA*sect	4	0.20	0.05	0.30	0.8791
	FV	TIA	2	214.12	107.08	13.33	4.156e-06
		sect	2	995.85	497.92	62.00	< 2e-16
		TIA*sect	4	83.92	20.98	2.61	0.0372
L. botrana	TA	TIA	4	401	100.16	16.97	6.640e-14
		sect	2	2269	1134.64	192.23	< 2.2e-16
		TIA*sect	8	465	58.16	9.85	8.843e-14
	CA	TIA	4	542	135.39	33.66	< 2.2e-16
		sect	2	397	198.73	49.40	< 2.2e-16
		TIA*sect	8	282	35.23	8.76	4.951e-12
	DA	TIA	4	392	97.99	24.07	< 2.2e-16
		sect	2	5962	2981.21	732.37	< 2.2e-16
		TIA*sect	8	256	32.01	7.86	1.29e-10
	GS	TIA	4	11141	2785	87.51	< 2.2e-16
		sect	2	70682	35341	1110.40	< 2.2e-16
		TIA*sect	8	1650	206	6.48	1.851e-08
	AS	TIA	4	4465	1116.40	66.34	< 2.2e-16
		sect	2	45192	22596.00	1342.73	< 2.2e-16
		TIA*sect	8	671	83.8	4.98	3.446e-06
	T	TIA	4	0.83	0.21	1.91	0.1091
		sect	2	5.23	2.61	24.17	2.022e-10
		TIA*sect	8	0.72	0.09	0.83	0.5744
	IT	TIA	4	298.00	74.40	11.59	2.257e-09
		sect	2	6679.00	3339.70	520.35	< 2.2e-16
		TIA*sect	8	78.00	9.80	1.53	0.1420
	FL	TIA	4	160.10	40.00	0.53	0.7163
		sect	2	29474.20	14737.10	193.87	< 2.2e-16
		TIA*sect	8	618.00	77.30	1.02	0.4237
	FD	TIA	4	1.34	0.33	1.01	0.4021
		sect	2	80.06	40.03	121.15	< 2.2e-16
		TIA*sect	8	1.96	0.24	0.74	0.6566
	FV	TIA	4	150.83	37.71	4.22	0.0025
		sect	2	1498.44	749.22	83.92	< 2.2e-16
		TIA*sect	8	32.12	4.02	0.45	0.8903

[a] Parameter: TA= Track angle (degrees); CA = Course angle (degrees); DA = Drift angle (degrees); GS = Ground speed (mm/s); AS = Air speed (mm/s); T = Turns/second (Hz); IT = Intern-turns (mm); FL = Flight length (mm); FD = Flight duration (s); FV = Flight velocity (mm/s)

[b] Variable: TIA = thiacloprid dose; sect = wind tunnel section; TIA*sect = interaction between TIA andflight track section

Table S5. ANOVA results for the effect of species and wind tunnel section on flight track parameters. Means, SEMs and significant differences shown in Figure 2.

Parameter	Variable [b]	Df	Sum Sq	Mean Sq	F	Pr(>F)
TA	sp	2	45	22.49	3.84	0.0215
	sect	2	1076	538.18	91.88	< 2.2e-16
	sp*sect	4	535	133.71	22.83	< 2.2e-16
CA	sp	2	831	415.53	96.23	< 2.2e-16
	sect	2	168	83.96	19.44	3.701e-09
	sp*sect	4	620	155.10	35.92	< 2.2e-16
DA	sp	2	1431	715.69	197.25	< 2.2e-16
	sect	2	3026	1513.01	416.99	< 2.2e-16
	sp*sect	4	179	44.79	12.34	5.086e-10
GS	sp	2	43813	21906.50	613.92	< 2.2e-16
	sect	2	47272	23636.10	662.39	< 2.2e-16
	sp*sect	4	6124	1531.10	42.91	< 2.2e-16
AS	sp	2	20920	10460.20	502.77	< 2.2e-16
	sect	2	32780	16389.80	787.77	< 2.2e-16
	sp*sect	4	3687	921.70	44.30	< 2.2e-16
T	sp	2	34.84	17.42	19.73	1.947e-08
	sect	2	54.87	27.43	31.08	3.070e-12
	sp*sect	4	12.39	3.10	3.51	0.0088
IT	sp	2	11776	5887.80	817.64	< 2.2e-16
	sect	2	14113	7056.30	979.90	< 2.2e-16
	sp*sect	4	1670	417.50	57.98	< 2.2e-16
FL	sp	2	207.00	103.50	1.50	0.2269
	sect	2	15780	7890.00	114.08	< 2.2e-16
	sp*sect	4	955.20	238.80	3.45	0.0096
FD	sp	2	5.52	2.76	11.54	1.988e-05
	sect	2	29.21	14.61	61.05	< 2.2e-16
	sp*sect	4	1.05	0.26	1.10	0.3606
FV	sp	2	451.92	225.96	27.45	4.573e-11
	sect	2	821.96	410.98	49.93	< 2.2e-16
	sp*sect	4	97.15	24.29	2.95	0.0217

[a] Parameter: TA= Track angle (degrees); CA = Course angle (degrees); DA = Drift angle (degrees); GS = Ground speed (mm/s); AS = Air speed (mm/s); T = Turns/second (Hz); IT = Intern-turn distance (mm); FL = Flight distance (mm); FD = Flight duration (s); FV = Flight velocity (FL/FD, $mm \cdot s^{-1}$)

[b] Variable: sp = species; sect = wind tunnel section; sp*sect = interaction among variables sp and sect

Table S6. ANOVA result for the effect of TIA and pheromone concentration on EAG responses. Means and SEMs shown in Figure 3. For models where both Pheromone and TIA were significant, the interaction was tested and found to be not significant. Refer to Supplementary Figure S5 for a description of the EAG parameters.

Species	EAG parameter	Variable	Df	Sum Sq	Mean Sq	F	Pr(>F)
C. pomonella	Downward phase	Pheromone	4	826970	206743	70.48	<2e-16
		TIA	4	15441	3860	1.32	0.2630
	Maximum response	Pheromone	4	191.41	47.85	409.01	<2e-16
		TIA	4	0.23	0.06	0.48	0.7504
	Upward phase	Pheromone	4	51835827	12958957	292.55	<2e-16
		TIA	4	45620	11405	0.26	0.9051
G. molesta	Downward phase	Pheromone	4	1053974	263494	93.58	<2e-16
		TIA	4	8294	2074	0.74	0.5674
	Maximum response	Pheromone	4	234.06	58.52	382.99	<2e-16
		TIA	4	1.36	0.34	2.23	0.0646
	Upward phase	Pheromone	4	14087508	3521877	158.15	<2e-16
		TIA	4	326779	81695	3.67	0.0059
L. botrana	Downward phase	Pheromone	4	331489	82872	36.39	<2e-16
		TIA	4	5952	1488	0.65	0.6247
	Maximum response	Pheromone	4	176.07	44.02	408.53	<2e-16
		TIA	4	3.25	0.81	7.55	6.658e-06
	Upward phase	Pheromone	4	16509914	4127479	285.61	<2e-16
		TIA	4	203104	50776	3.51	0.0077

Table S7. Pairwise comparisons among TIA doses for the statistical models in Supplementary Table S6 in which the effect of TIA was significant (ANOVA, P < 0.05). The comparisons were done with both, all the pheromone doses or with each pheromone dose individually, if the former was significant. In *G. molesta* the significant effect of TIA on the upward phase disappeared when comparing each pheromone concentration individually. In *L. botrana* the effect of TIA on maximum response holds when comparing each pheromone concentration individually, but the significant model effect of TIA on the upward phase does not result in significant pairwise differences among TIA doses. Different letters in a given row indicate significant differences among TIA doses (Tukey´s test, P< 0.05).

**Species**	**EAG Parameter**	**Pheromone concentration**	**TIA dose**				
			**Acetone**	**$LC_{0.001}$**	**LC_{1}**	**LC_{10}**	**LC_{20}**
G. molesta	Upward phase	All	a	ab	b	ab	ab
		n-hexane	a	a	a	a	a
		1 ng	a	a	a	a	a
		10 ng	a	a	a	a	a
		100 ng	a	a	a	a	a
		1 µg	a	a	a	a	a
L. botrana	Maximum response	All	a	a	b	a	a
		n-hexane	ab	a	b	a	a
		1 ng	ab	a	b	a	a
		10 ng	ab	a	b	a	a
		100 ng	ab	a	b	a	a
		1 µg	ab	a	b	a	a
	Upward phase	All	a	a	a	a	a
